# Music-based interventions for acute and short-term anxiety in nursing students: a systematic review and meta-analysis

**DOI:** 10.3389/fpubh.2026.1974164

**Published:** 2026-10-26

**Authors:** Bin Chen, Shanshan Wu, Zhuan Zou, Hu Gao

**Affiliations:** 1 Department of Emergency, West China Second University Hospital, Sichuan University, Chengdu, China; 2 Key Laboratory of Birth Defects and Related Diseases of Women and Children, Sichuan University, Ministry of Education, Chengdu, China; 3 Department of Emergency, WCSUH–Tianfu Sichuan Provincial Children's Hospital, Meishan, China

**Keywords:** anxiety, music listening, music therapy, nursing students, systematic review, meta-analysis, health workforce

## Abstract

**Background:**

Nursing students encounter acute anxiety during examinations, simulations, clinical skills training, and other health-related student contexts. We evaluated music-based interventions, with acute or short-term self-reported anxiety as the principal outcome and other psychological, physiological, health, and educational outcomes as secondary domains.

**Methods:**

Following peer review, we repeated searches in PubMed/MEDLINE, CINAHL Complete, Embase, APA PsycINFO, the Cochrane Central Register of Controlled Trials (CENTRAL), the Web of Science Core Collection, and ClinicalTrials.gov from their inception to 19 September 2026, without outcome, study design, publication type, or language limits. Two reviewers independently screened, extracted, and assessed the risk of bias. The principal meta-analysis used a uniform post-intervention Hedges’ *g* from each eligible, individually randomized parallel-group trial. Random-effects models were estimated using Restricted Maximum Likelihood or Residual Maximum Likelihood (REML); Wald confidence intervals were the primary estimate, and Hartung–Knapp and prediction intervals were used to assess small-sample uncertainty.

**Results:**

Of the 737 records identified, 337 duplicates were removed, and 400 records were screened. A total of 64 reports were sought; 39 unique complete documents were assessed; 26 reports representing 24 studies were included; and 13 full-text reports were excluded. Twenty studies were randomized, and four were non-randomized. Nine parallel-group trials (650 participants) contributed to the principal anxiety synthesis (*g* = −0.31, 95% confidence interval (CI): −0.58 to −0.04; *p* = 0.024; *I*^2^ = 64%; *τ*^2^ = 0.107). The Hartung–Knapp interval (from −0.63 to 0.01; *p* = 0.055) and the 95% prediction interval (from −1.15 to 0.53) crossed zero. All nine principal trials were at high overall risk of bias, and certainty was very low.

**Conclusion:**

Music-based interventions may reduce acute or short-term self-reported anxiety on average. However, the results are sensitive to the method used to quantify uncertainty, and the effects are likely to vary across settings. Evidence is insufficient to infer sustained psychological benefits, improved learning, or support for routine implementation.

**Systematic review registration:**

The review was registered in PROSPERO (CRD420251274752). Public record URL: https://www.crd.york.ac.uk/PROSPERO/view/CRD420251274752.

## Introduction

Investing in nursing education is a health-workforce priority because nurses are central to health service delivery and progress toward universal health coverage (1). Nursing students encounter examinations, simulations, invasive-skills training, and early clinical placements while managing workloads, responsibilities, observations, and fears of making errors (2–5). Anxiety and related distress are common across nursing programs (6–9). Support for students’ mental health is therefore relevant to both their participation in training and the future nursing workforce.

In this review, music-based intervention is an umbrella term, not a claim of equivalence. We distinguish passive music listening or music medicine, active music making, and therapist-delivered music therapy. The last category requires a structured therapeutic intervention delivered by a music therapist; researcher- or educator-delivered listening is not labeled as formal music therapy (10, 11). We also identify trials in which music was added to a shared co-intervention, such as aromatherapy, because these comparisons estimate only the incremental contribution of music.

Music may redirect attention away from threat cues, support emotion regulation, increase perceived control when choice is offered, and alter autonomic arousal (12–17). The same attentional mechanism may also impose cognitive load or obscure task-relevant auditory information. Listening before a stressor may therefore differ from listening during simulation, skills performance, assessment, or clinical activity, where distraction could impair instruction, communication, alarms, or performance. These mechanisms were proposed more often than they were measured across the included studies.

Earlier reviews addressed test anxiety and performance in nursing students (18, 19). A 2026 meta-analysis across health sciences programs included 24 nursing-student reports (20). After conducting a corrective search and full-text reassessment, 15 of the 24 reports were included in the present review (resulting in a 62.5% overlap within each review, with a Jaccard overlap of 45.5% across 33 unique reports). The contribution of this review is therefore a nursing-specific, outcome-free update with design verification, separate analyses of randomized and non-randomized evidence, unit-of-analysis safeguards, an explicitly defined principal anxiety estimand, small-sample sensitivity analyses, and the Grading of Recommendations Assessment, Development and Evaluation (GRADE), rather than a largely independent evidence base.

The primary review question is as follows: among nursing students, do passive music listening, active music making, structured therapist-delivered music therapy, or music-based educational interventions, compared with no music, usual activity, an attention or sensory control, or a matched co-intervention, reduce acute or short-term self-reported anxiety at the first eligible post-intervention assessment? Other psychological, physiological, student health, and educational outcomes were secondary domains and were not interpreted as evidence that reduced anxiety improves competence or sustains mental health.

## Methods

### Protocol and reporting

The protocol was prospectively registered in PROSPERO (CRD420251274752), and the review was reported in accordance with the PRISMA 2020 guidelines (21). In response to peer review, we revised the electronic search by removing outcome and study-design restrictions, while retaining acute or short-term anxiety as the principal outcome. The amendment, search date, and outcome-selection hierarchy were documented before the corrective search was conducted.

### Eligibility criteria and outcome domains

Participants were nursing students or student nurses at any stage of preregistration education. Eligible interventions included passive music listening or music medicine, active music making, structured therapist-delivered music therapy, and a music-based educational intervention. Music added to a co-intervention was eligible only when a comparator receiving the same co-intervention isolated the incremental music component. Studies using music solely as a metronomic or mnemonic cue were excluded if the intervention did not target psychological, physiological, student health, or broader educational outcomes. Comparators included no music, silence, usual education or activity, attention or sensory controls, and matched active controls.

The principal outcome was acute or short-term, self-reported state or situational anxiety at the first eligible post-intervention assessment. The context could occur during an examination, a clinical-skill simulation, an early clinical activity, or a student health event. Trait anxiety and test-specific anxiety were reported separately when they did not align with the selected acute construct. Stress, mood, burnout, pain, sleep, comfort, physiological measures, biomarkers, self-efficacy, knowledge, skills performance, and academic outcomes were secondary domains. Randomized and non-randomized controlled studies were eligible to be included; uncontrolled studies, reviews, editorials, case reports, and reports without an eligible controlled comparison were excluded.

### Information sources and search strategy

We searched PubMed/MEDLINE, CINAHL Complete, Embase, APA PsycINFO, the Cochrane Central Register of Controlled Trials, the Web of Science Core Collection, and ClinicalTrials.gov from their inception through 19 September 2026. The corrective strategies combined only music-based interventions and nursing-student concepts. No outcome, study design, publication type, or language filters were applied. Complete platform-specific syntax, execution dates, and retrieval counts are provided in Supplementary Table S1.

The seven sources provided a total of 737 records: 114 from PubMed/MEDLINE, 191 from CINAHL Complete, 167 from Embase, 72 from CENTRAL, 36 from APA PsycINFO, 133 from the Web of Science Core Collection, and 24 from ClinicalTrials.gov. Database exports were preserved and deduplicated at the report level using DOI or registry identifiers, exact bibliographic matching, and a manual review of similar entries. Reference and companion-report checks supplemented electronic retrieval. The corrective search was a post-peer-review amendment and is reported accordingly.

### Study selection and data extraction

Two reviewers independently screened 400 titles and abstracts and 39 unique full documents; they extracted study and outcome data, and resolved disagreements by discussion; a third reviewer was not required. Of the 64 reports sought, 23 could not be retrieved as complete documents, one was available only as a conference abstract, and one was an exact duplicate. Reports were linked to study families using authors, setting, sample, recruitment dates, intervention, outcomes, registry identifiers, and companion publication information. We did not impute unpublished numerical outcome data.

When several anxiety measures were eligible, the hierarchy was as follows: the study-designated primary acute anxiety outcome; a validated state or situational measure; or a test- or event-specific anxiety measure. We selected the first postintervention assessment and did not count any participant twice. Trait anxiety and stress were not used as substitutes for the principal anxiety construct (22, 23). We selected the first postintervention assessment and did not count the same participants twice. Accordingly, trait anxiety and stress were not used as substitutes for the principal anxiety construct. In multi-arm trials, the comparison that isolated music was selected; for Öner et al. and Son et al., aromatherapy plus music was compared with aromatherapy alone.

### Risk of bias assessment

Two reviewers independently assessed each target result using Risk of Bias 2 (RoB2), including the cluster and crossover variants where applicable, or Risk of Bias in Non-Randomized Studies of Interventions (ROBINS-I) (24–26). The target effect was assignment to the music-based intervention for the selected outcome. Prespecified confounders for non-randomized studies included baseline outcome severity, age, and sex; academic level or prior performance; musical experience or preference; and concurrent stress-management activities. Domain judgments and rationales are reported in Supplementary Tables S4, S5.

### Reporting bias assessment

Funnel plots and regression tests were performed only when at least 10 sufficiently comparable studies were available (27). No synthesis met this threshold. Therefore, reporting bias was judged as undetermined and was not downgraded solely because formal testing was unavailable.

### Certainty of evidence

We used GRADE to assess the risk of bias, inconsistency, indirectness, imprecision, and publication bias (28). Publication bias was downgraded only when independent evidence supported a concern, not merely because fewer than 10 studies were available.

### Data synthesis

The principal synthesis included one effect from each individually randomized parallel-group trial that reported usable postintervention arm summaries. Cluster-randomized, crossover, and non-randomized studies were not pooled in this model unless valid design-adjusted effects and standard errors (SEs) were available. Hedges’ *g* standardized across anxiety instruments (29). For two independent groups, *g* = Jd, where *J* = 1–3/(4df − 1); its variance was (n1 + n0)/(n1n0) + g^2^/(2df). When only a symmetric 95% confidence interval was available, SE was reconstructed as (upper − lower)/(2 × 1.959964).

Random-effects models used inverse-variance weights wi = 1/(SEi^2^ + *τ*^2^), with *τ*^2^ estimated by restricted maximum likelihood. The pooled estimate was Σwiyi/Σwi. The primary 95% confidence interval used the normal quantile. An unmodified Hartung–Knapp sensitivity interval used q* = Σwi(yi − *μ*)^2^/(*k* − 1), SEHK = √(q*/Σwi), and a t distribution with *k* − 1 degrees of freedom. Heterogeneity was described with *Q*, *I*^2^, *τ*^2^, and a 95% prediction interval was also calculated (29, 30). The analyses used Python 3.12.13, NumPy 2.3.5, SciPy 1.17.0, and Matplotlib 3.10.8 (31).

The principal estimand was deliberately uniform: the unadjusted postintervention standardized mean difference across all included trials. As a sensitivity analysis, post-minus-pre–change-score effects were calculated for eight trials with compatible baseline and follow-up data. When a change-score SD was not reported, SDchange = √(SDpre^2^ + SDpost^2^ − 2*r*·SDpre·SDpost), using *r* = 0.50 primarily and *r* = 0.25 and 0.75 in sensitivity analyses. We also examined passive listening, the exclusion of add-on interventions, standalone passive listening, the exclusion of the high-risk trial from the randomization domain, and leave-one-out analyses.

The crossover trial by Lai et al. lacked paired variance or first-period data. The two cluster-randomized trials lacked sufficient cluster-adjusted information. These studies were not included in the principal pool. Stress, dysmenorrhea-related pain, physiological outcomes across different stressors, and educational constructs were not pooled because the number of comparable studies was small and clinical or methodological heterogeneity was substantial. Non-randomized effects were presented separately.

## Results

### Study selection

The corrective search identified 737 records. After 337 duplicates were removed, 400 titles and abstracts were screened, and 336 of these were excluded. A total of 64 reports were sought; 23 could not be retrieved. Of the 41 retrieved records, one was an abstract-only report, and one was an exact duplicate, leaving 39 unique, complete documents for full-text assessment. A total of 13 were excluded, and 26 reports representing 24 independent studies were included. Nine individually randomized parallel-group trials contributed to the principal anxiety meta-analysis (Figure 1; Supplementary Tables S6, S7).

**Figure 1:**
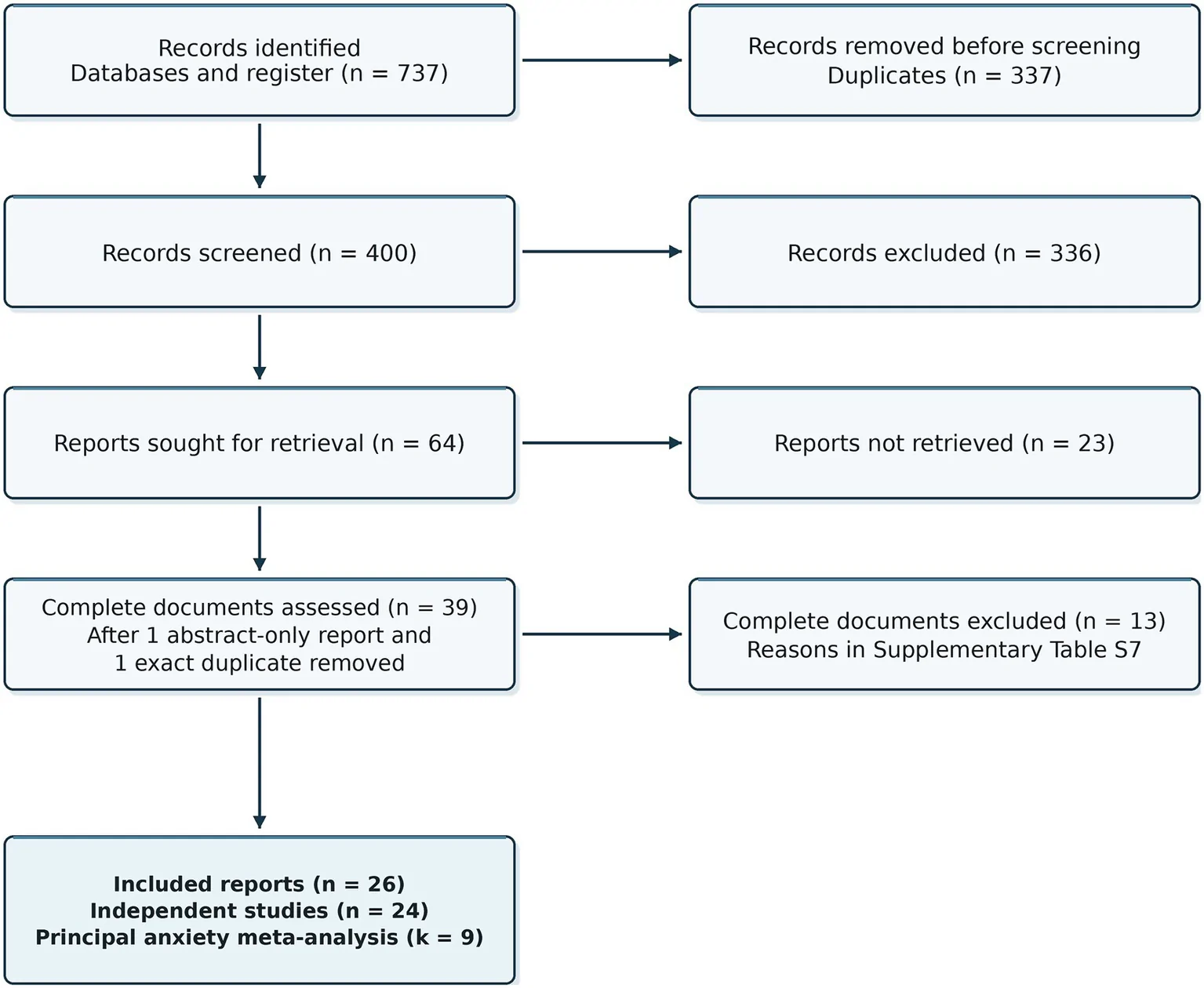
PRISMA 2020 flow diagram for the corrective search. Of the 737 records, 337 duplicates were removed; 400 records were screened; 64 reports were sought; 39 unique complete documents were assessed; 26 reports were included; and these reports represented 24 studies.

### Study and intervention characteristics

The 24 studies comprised 20 randomized studies—17 individually randomized parallel-group trials, two cluster-randomized trials, and one crossover trial—and four non-randomized controlled studies (32–57). A total of 20 articles evaluated passive or background listening, two evaluated active music-making interventions, one structured group intervention delivered by a music therapist, and one music-based auditory-training intervention. Comparators included silence, no music or usual activity, sensory or attention controls, and matched co-interventions. Intervention selection, provider, delivery, dose, tempo, and context are detailed in Supplementary Tables S2, S3.

Twelve studies were conducted in Türkiye; the remaining studies were conducted in the United States, Iran, Taiwan, the Philippines, Austria, India, and South Korea. Sex reporting was incomplete, but women formed the large majority in studies with usable data. No included study was conducted in Africa, Latin America, or Oceania. The evidence does not support reliable sex, gender, cultural, geographic, or equity analyses.

### Risk of bias

Among the 20 randomized studies, 18 were judged to be at a high overall risk of bias, and two had some concerns; none was at a low overall risk. All four non-randomized studies were at a serious overall risk of bias. Common concerns were inadequate allocation or cluster handling, missing data, unblinded self-reported outcomes, selective reporting, and uncontrolled class, period, expectancy, or attention effects (Figures 2, 3; Supplementary Tables S4, S5, Figure S1).

**Figure 2:**
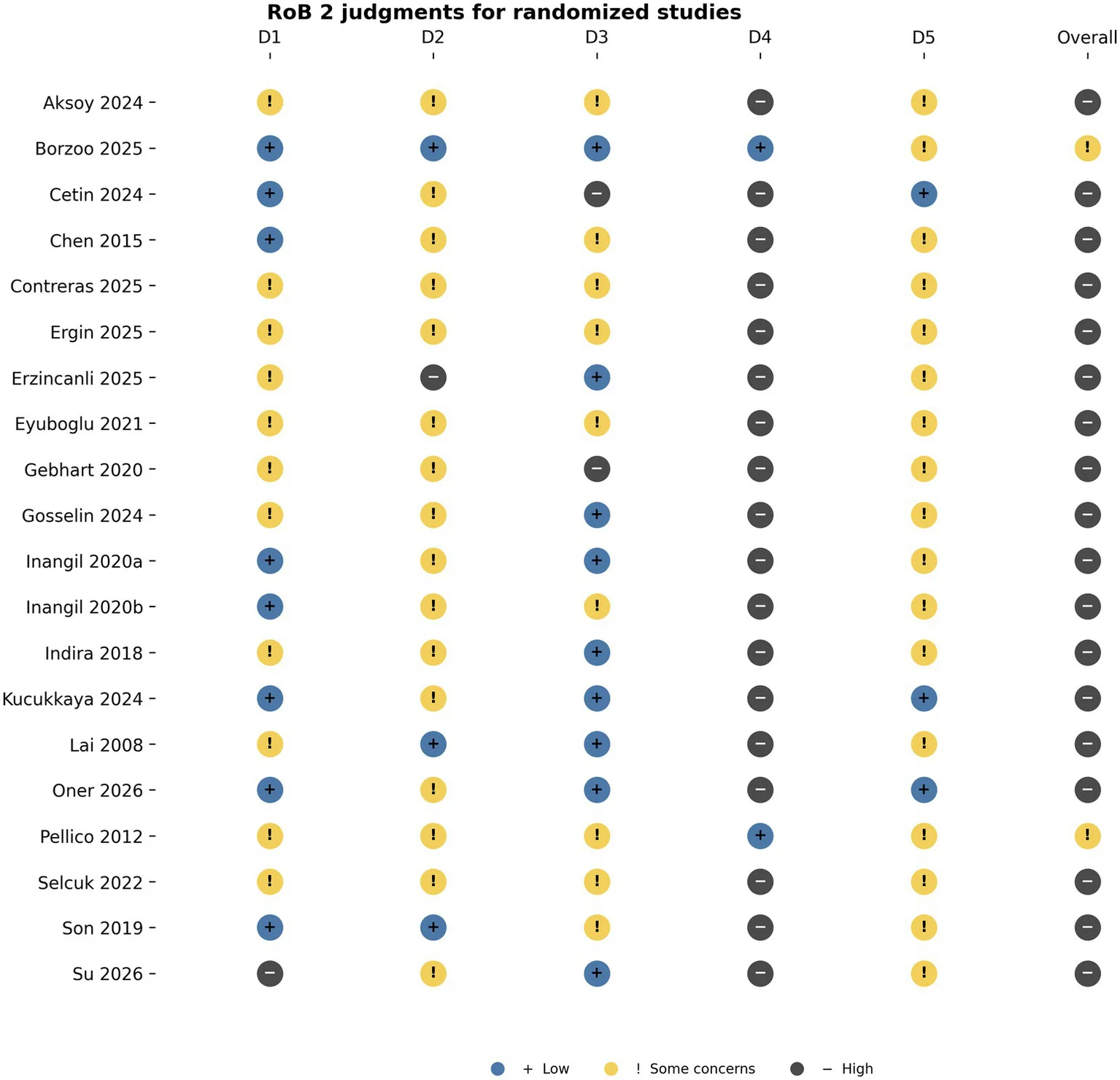
Risk-of-bias traffic-light plot for 20 randomized studies assessed using RoB 2 or the applicable cluster/crossover variant. D1, randomization process; D2, deviations from intended interventions; D3, missing outcome data; D4, outcome measurement; D5, selection of the reported result. The additional design domain for cluster and crossover trials is presented in Supplementary Table S4.

**Figure 3:**
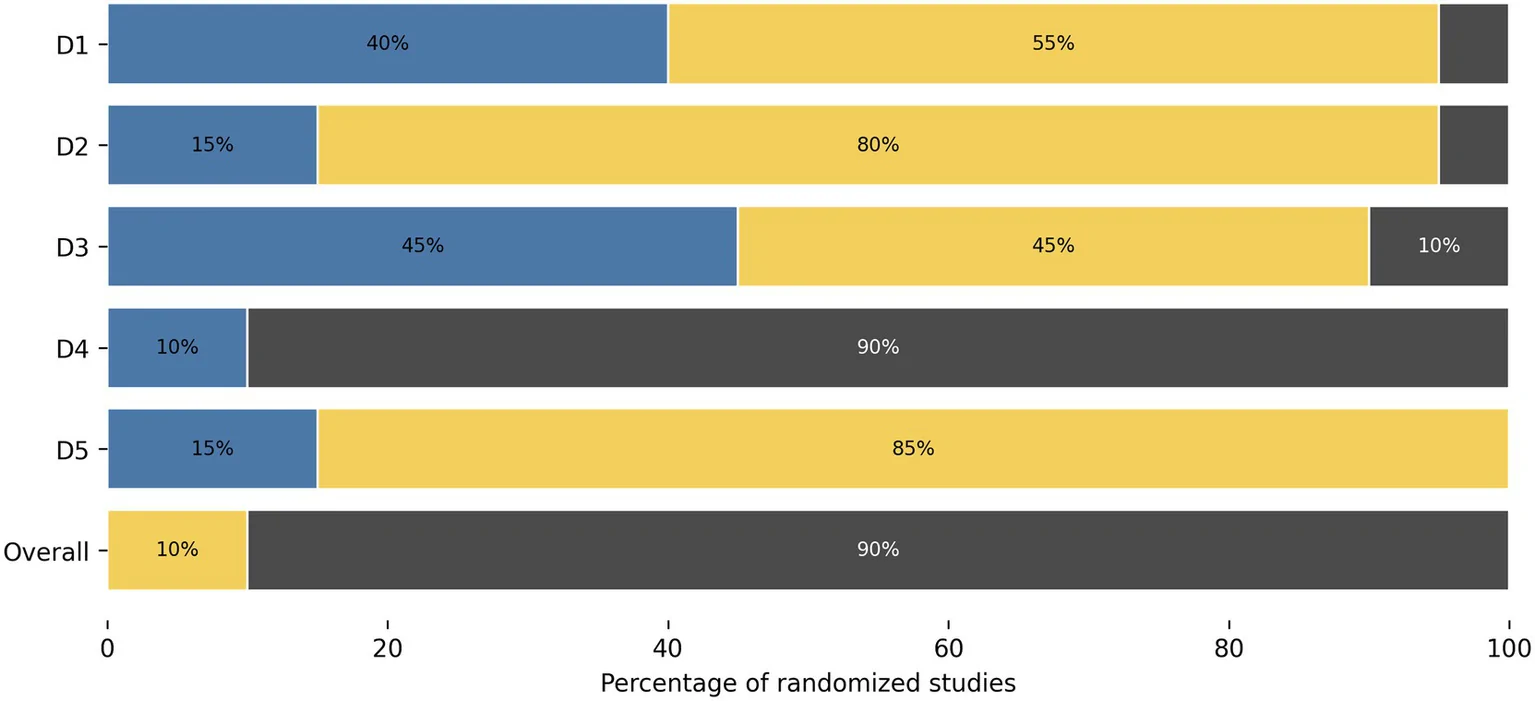
Distribution of RoB 2 judgments across 20 randomized studies. A total of 18 studies were at a high overall risk, two had some concerns, and none were at a low overall risk.

### Principal short-term anxiety outcome

Nine individually randomized parallel-group trials involving 650 participants contributed to the principal postintervention analysis. The REML estimate favored music-based intervention (Hedges’ *g* = −0.31, 95% CI: −0.58 to −0.04; *p* = 0.024; *I*^2^ = 64%; *τ*^2^ = 0.107; *Q* = 22.38, df = 8, *p* = 0.004). However, the Hartung–Knapp interval included no effect (−0.63 to 0.01; *p* = 0.055), as did the 95% prediction interval (−1.15 to 0.53) (Figure 4). All nine trials were at a high overall risk of bias.

**Figure 4:**
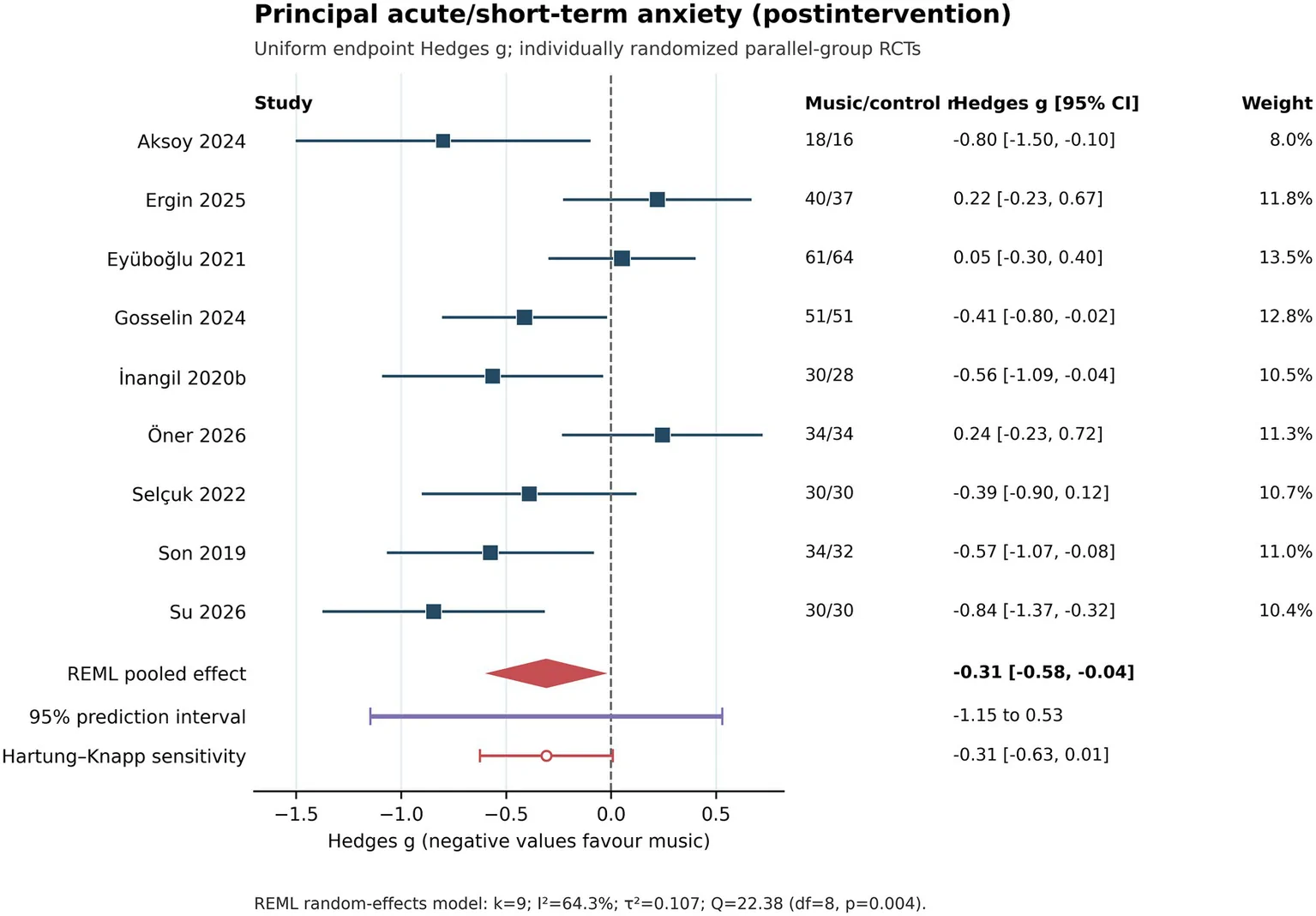
Random-effects meta-analysis of acute or short-term self-reported anxiety using one uniform postintervention Hedges’ *g* from each of nine individually randomized parallel-group trials. The diamond shows the REML estimate with the conventional 95% confidence interval. The Hartung–Knapp interval and prediction interval are reported below the plot. Negative values favor music-based interventions.

Excluding the structured active music-therapy trial yielded *g* = −0.36 (95% CI: −0.65 to −0.08; Hartung–Knapp CI − 0.71 to −0.02; *k* = 8). Excluding the two add-on contrasts gave *g* = −0.35 (−0.65 to −0.05; Hartung–Knapp CI − 0.72 to 0.02; *k* = 7). Standalone passive listening gave *g* = −0.43 (−0.75 to −0.12; Hartung–Knapp CI − 0.84 to −0.02; *k* = 6). Excluding the trial at high risk in the randomization domain gave *g* = −0.24 (−0.51 to 0.02; Hartung–Knapp CI − 0.56 to 0.08; *k* = 8). Leave-one-out Wald intervals crossed zero after the omission of six of the nine trials, indicating sensitivity to individual studies (Supplementary Tables S8–S9, Figures S2–S3).

Change-score analyses were available in eight trials. The estimates remained in the direction of lower anxiety at *r* = 0.25, 0.50, and 0.75, but their confidence intervals included no effect. These analyses used inferred correlations across several studies and excluded Gosselin et al. 2024 because compatible baseline summaries were unavailable. The principal effect corresponds to approximately 0.31 within-study standard deviations, but no validated, cross-instrument minimally important difference was identified for nursing students.

### Stress and student health outcomes

Stress results were not pooled. The randomized evidence came from menstrual stress and clinical skills contexts with markedly different magnitudes; the non-randomized lecture study was kept separate. These estimates do not support a common stress effect. Pain was not pooled either: three randomized studies evaluated primary dysmenorrhea using different intervention doses, timing, and analytical approaches. Study-specific effects and their sources are reported in Supplementary Table S10.

Other student health outcomes, including depressed mood, burnout, mood disturbance, comfort, and sleep-related outcomes, were sparse and heterogeneous. No common effect was estimated, and these outcomes were not used to support conclusions about acute anxiety, learning, or sustained mental health.

### Physiological outcomes

Physiological outcomes were reported across examination, simulation, clinical skills, and self-measurement settings. Due to differences in the measurement timing and stressors, no common effect was estimated. Directly verified arm-level effects are reported in Supplementary Table S10. Numerical values that could not be independently reproduced from the report-level data were summarized narratively and were not retained as quantitative effects.

### Educational outcomes

Educational outcomes included knowledge, self-efficacy, simulation or clinical skills performance, self-confidence, academic performance, and auditory diagnostic skills. Directly verified between-group results are presented in Supplementary Tables S10, S11. The majority of the estimates were imprecise or vulnerable to outcome-measure limitations. Gosselin et al. reported higher post-intervention self-efficacy (*g* = 0.73, 95% CI: 0.33–1.13) and higher instructor-rated performance in a selected subset of 20 participants (*g* = 1.71, 0.67–2.75); the subset result was particularly susceptible to selection bias. Su et al. found no clear difference in the PIVC checklist score (MD: 0.46, −2.35 to 3.27). Other constructs and time points were too dissimilar to pool.

Within-group improvement was not treated as evidence of efficacy. Some studies reported task-specific educational effects, including auditory discrimination, but measurement validity, assessor blinding, and delayed retention were inconsistently reported. The evidence does not establish that lowering self-reported anxiety improves knowledge, competence, grades, or retention.

### Reporting bias and certainty

No synthesis included at least 10 sufficiently comparable studies; reporting bias could therefore not be determined and was not used as a standalone downgrade. The certainty of the principal anxiety estimate was very low because all contributing trials were at a high risk of bias, heterogeneity was substantial, the contexts and intervention forms varied, and the Hartung–Knapp and prediction intervals included no effect. Secondary domains were also highly uncertain due to sparse, heterogeneous, or non-randomized evidence (Table 1).

**Table 1:** Summary of findings and certainty of evidence.

Outcome	Evidence	Effect	*I*^2^/*τ*^2^	HK interval	Prediction interval	Key limitations	Reporting bias	Certainty	Interpretation
Principal acute/short-term anxiety	9 parallel-group RCTs; 650 participants	*g* = −0.31 (95% CI: −0.58 to −0.04)	64% / 0.107	−0.63 to 0.01	−1.15 to 0.53	All nine trials at high RoB; varied interventions, controls, and contexts	Undetermined; not downgraded	Very low	A small average reduction is possible, but robust uncertainty and the expected range across settings include no effect.
Stress	2 RCTs plus 1 non-randomized study; not pooled	Study-specific estimates only	Not pooled	Not applicable	Not applicable	Different constructs and contexts; self-report; serious/high RoB	Undetermined	Very low	No common stress effect can be inferred.
Dysmenorrhea pain	3 RCTs; not pooled	Study-specific mean differences	Not pooled	Not applicable	Not applicable	Different timing, dose, and analytic basis; unblinded self-report	Undetermined	Very low	Pain findings are separate from the principal anxiety inference.
Physiological outcomes	7 heart-rate/pulse studies; 6 blood-pressure studies	Mixed study-specific effects	Not pooled	Not applicable	Not applicable	Different stressors and timing; some legacy reconstructed effects	Undetermined	Very low	No consistent physiological effect is established.
Educational outcomes	Construct-specific evidence; not pooled	Mixed knowledge, self-efficacy, skill, and performance findings	Not pooled	Not applicable	Not applicable	Non-equivalent constructs; assessor blinding, validity, and retention often unclear	Undetermined	Very low	Reduced anxiety cannot be assumed to improve learning or competence.

CI, confidence interval; *g*, Hedges’ standardized mean difference; HK, Hartung–Knapp; PI, prediction interval; RCT, randomized controlled trial; RoB, risk of bias. Publication bias was not downgraded solely because fewer than 10 comparable trials were available, as this precluded formal testing.

## Discussion

The average post-intervention estimate suggests a small reduction in acute or short-term self-reported anxiety, but this inference is fragile. The conventional Wald interval excluded zero, whereas the Hartung–Knapp interval narrowly crossed zero. The prediction interval spanned benefit, no benefit or harm, and the majority of the leave-one-out Wald intervals crossed zero. Together with a uniformly high risk of bias across the nine principal trials, these results support the possibility of efficiency rather than a definitive claim of efficacy.

The study-level comparison with the 2026 review shows meaningful overlap: 15 of the 24 reports were shared, while 9 were unique to each review. The present review differs in that it applies an outcome- and design-unrestricted corrective search, restricts the population to nursing students, verifies study design, separates non-randomized and design-invalid estimates, uses a uniform postintervention principal estimand, and quantifies small-sample uncertainty. Differences in effect size and certainty should therefore be attributed to differences in eligibility criteria, the inclusion of newly available studies, contrast selection, design handling, and analytical methods rather than to an independent evidence base (20). A detailed comparison is provided in Supplementary Table S12.

The observed reduction could reflect music-specific emotion regulation, attentional redirection, perceived control, or autonomic effects. Expectancy, demand characteristics, participant preference, and researcher attention are also plausible explanations for unblinded self-reported outcomes. The choice of comparator changes the causal question: silence and usual activity estimate the effect of adding music, sensory controls address a more specific contrast, and aromatherapy-only controls estimate the incremental contribution of music.

Reduced anxiety does not necessarily improve learning. Moderate arousal may support engagement, whereas excessive relaxation or auditory distraction may impair attention to instructions, communication, alarms, or task-relevant cues. Secondary psychological, physiological, student health, and educational findings were too heterogeneous for a common pooled interpretation. No included study formally evaluated cost, implementation fidelity, reach, equity, or scalability.

Intervention heterogeneity also limits generalization. The majority of the studies evaluated brief passive or background listening; active music making, therapist-delivered group work, and music-based auditory training involve different mechanisms, staff competencies, participant engagement, and dosage. Preference, familiarity, tempo, volume, delivery, duration, and prior musical experience were reported inconsistently, rendering them unsuitable for credible subgroup analysis or meta-regression.

Limitations include 23 reports for which a complete document could not be retrieved, potential selective non-publication, sparse evidence within intervention categories, and incomplete reporting on allocation, missing data, assessor blinding, adherence, and intervention fidelity. Although the corrective search used no language limit, non-English and grey literature may remain underrepresented. The principal outcome hierarchy was amended following peer review. To prevent the reuse of uncertain data, 38 ancillary estimates that could not be reproduced from arm-level report data and that were available only as rounded values from earlier forest plots were excluded from the quantitative dataset. Evidence was geographically concentrated and predominantly female.

Future trials should conceal allocation, use blinded performance assessors, prespecify a single validated acute anxiety outcome, separate from educational outcomes, and report arm-level data at every time point. Reports should distinguish passive listening, active music making, therapist-delivered therapy, and add-on designs; they should document preferences, familiarity, expectancy, distraction, adverse effects, fidelity, and access; and use adequate cluster or crossover analyses (58). Educational studies should use validated measures and delayed retention tests. Future updates should maintain outcome- and design-unrestricted searching.

## Conclusion

Music-based interventions may, on average, reduce acute or short-term self-reported anxiety among nursing students, but very-low-certainty in the evidence and sensitivity to uncertainty methods preclude definitive efficacy claims. The effects on learning, physiology, student health, and sustained wellbeing remain uncertain, and current evidence does not support the routine implementation of music-based interventions.

## Data Availability

The author-verified extraction workbook, risk-of-bias judgments, analysis inputs, statistical outputs, and figure-generation code are supplied in the Supplementary material. The principal estimate and every sensitivity analysis are reproducible from the accompanying arm-level files. Quantitative estimates that could not be independently reproduced from report-level data were excluded from the quantitative dataset.
