# Supplementary material for: Music-based interventions for acute and short-term anxiety in nursing students: a systematic review and meta-analysis
Source: Front Public Health. 2026 Oct 26;14:1974164. doi: 10.3389/fpubh.2026.1974164 (PMC13746330; doi:10.3389/fpubh.2026.1974164)
Supplement: Supplementary file 2 [file Supplementary_file_1.docx]

Supplementary material

Music-based interventions for acute and short-term anxiety in nursing students: a systematic review and meta-analysis

All searches were executed on 19 September 2026 without outcome, study-design, publication-type, or language restrictions. This supplement reports retrieval, study-level linkage, the author-verified principal analysis, and only those numerical secondary effects that could be reproduced directly from report-level data.

## Table S1A. Corrective search execution log

| **Source** | **Platform** | **Records** |
| --- | --- | --- |
| PubMed/MEDLINE | PubMed | 114 |
| CINAHL Complete | EBSCOhost | 191 |
| Embase | Embase.com | 167 |
| CENTRAL | Cochrane Library | 72 |
| APA PsycINFO | EBSCOhost | 36 |
| Web of Science Core Collection | Clarivate | 133 |
| ClinicalTrials.gov | ClinicalTrials.gov | 24 |
| Total |  | 737 |

## Table S1B. Complete copy-paste search strategies

| **Source** | **Final syntax** |
| --- | --- |
| PubMed/MEDLINE | ("Music"[Mesh] OR "Music Therapy"[Mesh] OR music*[tiab] OR singing[tiab] OR song*[tiab] OR drum*[tiab]) AND ("Students, Nursing"[Mesh] OR "nursing student*"[tiab] OR "student nurse*"[tiab] OR "nurse student*"[tiab] OR "nursing undergraduate*"[tiab] OR "undergraduate nursing"[tiab] OR "undergraduate nurs*"[tiab] OR "baccalaureate nurs*"[tiab] OR "prelicensure nurs*"[tiab] OR "pre-licensure nurs*"[tiab] OR "preregistration nurs*"[tiab] OR "pre-registration nurs*"[tiab] OR "nursing trainee*"[tiab] OR "nurse trainee*"[tiab] OR "nursing school*"[tiab] OR "school of nursing"[tiab] OR "schools of nursing"[tiab] OR "college of nursing"[tiab] OR "colleges of nursing"[tiab]) |
| CINAHL Complete | ((MH "Music+") OR (MH "Music Therapy+") OR TI music* OR AB music* OR TI singing OR AB singing OR TI song* OR AB song* OR TI drum* OR AB drum*) AND ((MH "Students, Nursing+") OR TI "nursing student*" OR AB "nursing student*" OR TI "student nurse*" OR AB "student nurse*" OR TI "nurse student*" OR AB "nurse student*" OR TI "nursing undergraduate*" OR AB "nursing undergraduate*" OR TI "undergraduate nurs*" OR AB "undergraduate nurs*" OR TI "baccalaureate nurs*" OR AB "baccalaureate nurs*" OR TI "prelicensure nurs*" OR AB "prelicensure nurs*" OR TI "pre-licensure nurs*" OR AB "pre-licensure nurs*" OR TI "preregistration nurs*" OR AB "preregistration nurs*" OR TI "pre-registration nurs*" OR AB "pre-registration nurs*" OR TI "nursing trainee*" OR AB "nursing trainee*" OR TI "nurse trainee*" OR AB "nurse trainee*" OR TI "nursing school*" OR AB "nursing school*" OR TI "school of nursing" OR AB "school of nursing" OR TI "college of nursing" OR AB "college of nursing") |
| Embase | ('music'/exp OR 'music therapy'/exp OR music*:ti,ab,kw OR singing:ti,ab,kw OR song*:ti,ab,kw OR drum*:ti,ab,kw) AND ('nursing student'/exp OR 'nursing student*':ti,ab,kw OR 'student nurse*':ti,ab,kw OR 'nurse student*':ti,ab,kw OR 'nursing undergraduate*':ti,ab,kw OR 'undergraduate nursing':ti,ab,kw OR 'undergraduate nurs*':ti,ab,kw OR 'baccalaureate nurs*':ti,ab,kw OR 'prelicensure nurs*':ti,ab,kw OR 'pre-licensure nurs*':ti,ab,kw OR 'preregistration nurs*':ti,ab,kw OR 'pre-registration nurs*':ti,ab,kw OR 'nursing trainee*':ti,ab,kw OR 'nurse trainee*':ti,ab,kw OR 'nursing school*':ti,ab,kw OR 'school of nursing':ti,ab,kw OR 'schools of nursing':ti,ab,kw OR 'college of nursing':ti,ab,kw OR 'colleges of nursing':ti,ab,kw) |
| CENTRAL | ([mh "Music"] OR [mh "Music Therapy"] OR (music* OR singing OR song* OR drum*):ti,ab,kw) AND ([mh "Students, Nursing"] OR (nurs* NEAR/3 (student* OR undergraduate* OR trainee* OR prelicen* OR preregistr*)):ti,ab,kw OR ("school of nursing" OR "schools of nursing" OR "college of nursing" OR "colleges of nursing"):ti,ab,kw) |
| APA PsycINFO | (DE "Music Therapy" OR TI music* OR AB music* OR TI singing OR AB singing OR TI song* OR AB song* OR TI drum* OR AB drum*) AND (TI "nursing student*" OR AB "nursing student*" OR TI "student nurse*" OR AB "student nurse*" OR TI "nurse student*" OR AB "nurse student*" OR TI "nursing undergraduate*" OR AB "nursing undergraduate*" OR TI "undergraduate nurs*" OR AB "undergraduate nurs*" OR TI "baccalaureate nurs*" OR AB "baccalaureate nurs*" OR TI "prelicensure nurs*" OR AB "prelicensure nurs*" OR TI "pre-licensure nurs*" OR AB "pre-licensure nurs*" OR TI "preregistration nurs*" OR AB "preregistration nurs*" OR TI "pre-registration nurs*" OR AB "pre-registration nurs*" OR TI "nursing trainee*" OR AB "nursing trainee*" OR TI "nurse trainee*" OR AB "nurse trainee*" OR TI "nursing school*" OR AB "nursing school*" OR TI "school of nursing" OR AB "school of nursing" OR TI "college of nursing" OR AB "college of nursing") |
| Web of Science Core Collection | TS=(music* OR singing OR song* OR drum*) AND TS=((nurs* NEAR/3 (student* OR undergraduate* OR trainee*)) OR "student nurse*" OR "nursing undergraduate*" OR "baccalaureate nurs*" OR "prelicensure nurs*" OR "pre-licensure nurs*" OR "preregistration nurs*" OR "pre-registration nurs*" OR "nursing school*" OR "school of nursing" OR "schools of nursing" OR "college of nursing" OR "colleges of nursing") |
| ClinicalTrials.gov | (music OR "music therapy" OR "music listening" OR singing OR drumming) AND ("nursing student" OR "nursing students" OR "student nurse" OR "student nurses" OR "nursing undergraduate" OR "nursing undergraduates" OR "prelicensure nursing" OR "pre-licensure nursing" OR "preregistration nursing" OR "pre-registration nursing") |

## Table S2. Included-study characteristics

| **ID** | **Study** | **Design** | **Total sample** | **Selected analytic sample** | **Academic level** | **Outcomes / instruments** | **DOI** |
| --- | --- | --- | --- | --- | --- | --- | --- |
| ST01 | Aksoy et al., 2024, Turkey | RCT | Randomized: 60; analyzed: 50 | Music 18; silent control 16 | First year | State anxiety; Vital signs; IMI skill score (STAI-S; HR, SBP, DBP; IMI skill score) | 10.1016/j.teln.2023.08.020 |
| ST02 | Bittman et al., 2004, USA | Nonrandomized crossover CCT | Total: 75; female: 64 | Not included in quantitative synthesis | First-year associate degree | Burnout; Mood states; Total mood disturbance (MBI; POMS) | 10.2202/1548-923x.1044 |
| ST03 | Borzoo et al., 2025, Iran | Individually randomized RCT | Total: 169; female: 87 | Music 56; control 53 | ≥ Second semester | Vital signs (HR, SBP, DBP) | 10.1038/s41598-025-97199-6 |
| ST04 | Çetin & Kaya, 2024, Turkey | RCT | Randomized: 168; analyzed: 129 | Music 42; control 45 | ≥ First year | Pain intensity; Comfort level (VAS; GCQ) | No DOI reported |
| ST05 | Chen et al., 2015, Taiwan | RCT | Randomized: 80; analyzed: 71 | Music 31; control 40 | College level | Depression level; Salivary cortisol (DMSRIA; ELISA) | 10.1111/ijn.12236 |
| ST06 | Contreras et al., 2025, USA | Cluster-randomized controlled trial (recording groups) | Randomized: 96; consented/completed: 89 | Music 49; control 40 | First semester | State anxiety; Vital signs (STAI-Y1; HR, BP) | 10.1097/01.NEP.0000000000001287 (companion report) |
| ST07 | Ergin et al., 2025, Turkey | RCT | Randomized: 81; analyzed: 77 | Music 40; control 37 | Second year | State anxiety; Comfort level; Vital signs; Learning satisfaction (SAI; VAS; SBP,DBP,HR; SSSCLQ) | No DOI reported |
| ST08 | Erzincanlı & Yüksel, 2025, Turkey | Cluster RCT (2 classes; 1 per arm) | Total: 73; female: NR | Excluded from pooling: one class per arm; no valid cluster-adjusted SE | First year | State anxiety; Vital signs (STAI-S; BP, pulse, saturation) | 10.14744/jern.2025.12316 |
| ST09 | Evangelista et al., 2017, Philippines | Nonrandomized CCT | Total: 42; female: NR | Music 21; control 21 | Undergraduate | Stress; Anxiety; Knowledge (DASS-Stress; DASS-Anxiety; quiz score) | 10.1515/ijnes-2016-0076 |
| ST10 | Eyüboğlu et al., 2021, Turkey | RCT | Randomized: 132; analyzed: 125 | Music therapy 61; control 64 | First year | State anxiety; Vital signs; OSCE skill scores (STAI; BP, pulse, respiration; OSCE skill scores) | 10.1016/j.nedt.2020.104687 |
| ST11 | Gebhart et al., 2020, Austria | Individually randomized four-arm parallel-group RCT | Randomized: 72 (18/arm); completed: 57 | Body percussion 14; uninstructed free-hour control 15 | Nursing students preparing for a written examination | State anxiety; visual analogue stress; salivary cortisol; salivary IgA | 10.1111/ijn.12788 |
| ST12 | Gosselin et al., 2024, USA | RCT | Randomized/analyzed: 102 | Music 51; control 51 | Third year | State anxiety; Self-efficacy; Simulation performance (6-item STAI; CSGSE; instructor rating 0-100) | 10.1016/j.ecns.2024.101625 |
| ST13 | İnangil et al., 2020a, Turkey | RCT | Total: 90; female: 79 | Not included in quantitative synthesis | Nursing students | State anxiety; Vital signs (STAI-S; BP, pulse, SpO2) | 10.1016/j.eujim.2019.101041 |
| ST14 | İnangil et al., 2020b, Turkey | RCT | Randomized: 90; analyzed: 86 | Music 30; control 28 | Before first clinical practice | State anxiety; Trait anxiety (STAI-S; STAI-T) | 10.5152/fnjn.2020.19075 |
| ST15 | Ince & Çevik, 2017, Turkey | Nonrandomized CCT | Total: 73; female: 54 | Music 34; control 39 | First year | State anxiety; Vital signs; Blood draw skill steps (STAI-S; BP, pulse) | 10.1016/j.nedt.2017.02.009 |
| ST16 | Indira et al., 2018, India | Individually randomized RCT | Total: 191; female: 191 | Not included in quantitative synthesis | First year | Academic performance (19-item Academic Performance Rating Scale) | 10.4103/ijam.ijam_32_18 |
| ST17 | Küçükkaya & Başgöl, 2024, Turkey | RCT | Total: 96; female: 96 | Music 32; control 32 | Nursing students | Pain intensity; Perceived stress; Menstrual symptoms (100mmVAS; PSS; MSS) | 10.1186/s12905-024-03398-0 |
| ST18 | Lai et al., 2008, Taiwan | Randomized crossover trial | Total: 38; female: 38 | Excluded from pooling: paired variance/first-period data unavailable | College level | Examination anxiety; State anxiety; Pulse rate; Finger temperature (TAI; STAI) | 10.1016/j.nedt.2008.05.011 |
| ST19 | Öner et al., 2026, Turkey | RCT | Total: 102; female: 68 | Aromatherapy + music 34; aromatherapy alone 34 | First year | Anxiety; Self-esteem; Pulse; SBP; DBP (STAI; RSE; Pulse and BP measurements) | 10.3928/01484834-20260129-02 |
| ST20 | Pellico et al., 2012, USA | Individually randomized parallel-group RCT | Total: 78; female: 94% | ITT n=78 for principal scores using LOCF; complete-case posttest n=69 | First-semester accelerated master's-entry nursing students | Organ identification; diagnosis of heart, lung, and bowel sounds (25-item auditory assessment) | 10.5480/1536-5026-33.4.234 |
| ST21 | Karakus Selcuk & Baysal, 2022, Turkey | Individually randomized three-arm parallel-group RCT | Randomized: 90; completed: 84; all female | Music 30; control 30 in reported ITT tables (complete cases: 25 and 29) | First- to fourth-year undergraduate nursing students | Menstrual pain intensity; state anxiety (10-cm VAS; STAI-S) | 10.1016/j.eujim.2022.102192 |
| ST22 | Son et al., 2019, South Korea | RCT | Randomized: 107; analyzed: 98 | Aromatherapy plus music 34; aromatherapy 32 | Second year | Test anxiety; State anxiety; Stress; Foley catheterization skill score (RTA; STAI-K; NRS; Foley catheterization skill score) | 10.3390/ijerph16214185 |
| ST23 | Su et al., 2026, Turkey | Individually randomized parallel-group RCT | Analyzed: 60; report flow shows an unresolved 144-to-60 discrepancy | Music 30; control 30 | First-year nursing students, spring semester | State anxiety; self-confidence; PIVC skill; pulse; respiration; SBP; DBP; SpO2 | 10.4274/mnm.2025.25400 |
| ST24 | Summers et al., 1990, USA | Nonrandomized controlled trial using two intact course cohorts | Total: 45; female: 42 | Music 21; control 24 | Junior baccalaureate nursing students | State anxiety; test anxiety; pulse rate (STAI-S; Test Anxiety Inventory; self-measured pulse) | 10.3928/0148-4834-19900201-06 |

## Table S3. Intervention taxonomy and delivery

| **Study** | **Category** | **Duration** | **Sessions** | **Provider** | **Music selection** | **Delivery** | **Context** | **Comparator** |
| --- | --- | --- | --- | --- | --- | --- | --- | --- |
| Aksoy 2024 | MM | 5 min | 1 | Researcher / study team | Self-selected pop music | Individual listening via headphones/earphones | Examination / OSCE | Silent environment |
| Bittman 2004 | MT | 60 min | 6 weekly | Trained facilitators | HealthRHYTHMS group empowerment drumming | Group sessions with drums and percussion instruments | Early clinical education | Usual academic routine |
| Borzoo 2025 | MM | 20 min | 1 | Researcher / study team | Classical / instrumental music | Free-field playback | Examination / OSCE | Placebo (cotton with 10 drops of water) |
| Cetin 2024 | MM | 30 min | 2 | Researcher / study team | Non-verbal music / nature sounds | Individual listening via headphones/earphones | Menstrual period | No intervention / routine care |
| Chen 2015 | MM | 40 min | 20 | Researcher / study team | Chinese five-element music | Free-field playback via CD player | NR | Routine lifestyle (no music therapy) |
| Contreras 2025 | MM | 15 min | 1 | Researcher / study team | Classical / instrumental music | Free-field playback | NR | Sitting quietly (no music) |
| Ergin 2025 | MM | 30 min | 1 | Researcher / study team | Classical / instrumental music | Free-field playback | Simulation | No music intervention |
| Erzincanli 2025 | MM | Single session | 1 | Researcher / study team | Traditional Turkish / maqam music | Playback via portable computer | Clinical skill practice | Standard skills training (no music) |
| Evangelista 2017 | MM | 3 hour | 1 | Researcher / study team | Classical / instrumental music | Free-field playback | Lecture | Regular lecture (no music) |
| Eyuboglu 2021 | MT | 60 min | 5 | Music therapist | Music-based intervention | NR | Examination / OSCE | Routine teaching (no music therapy) |
| Gebhart 2020 | Active music intervention / body percussion | 45-60 min | Familiarization plus two measured sessions | Professional music therapist | Structured body-percussion exercises | Group session | Normal school day and immediately before a written examination | Uninstructed free hour |
| Gosselin 2024 | MM | 15 min | 1 | Researcher / study team | Classical / instrumental music | Individual listening via headphones/earphones | Simulation | Normal preparation (no music) |
| Inangil 2020a | MM | 15 min | 1 | Researcher / study team | Traditional Turkish / maqam music | Individual listening via MP3 player | Examination / OSCE | Free time (no intervention) |
| Inangil 2020b | MM | 15 min | 6 (3/week for 2 weeks) | Researchers/instructors | Traditional Turkish Mahur instrumental music | Portable computer in a quiet, dimly lit room | Before first clinical practice | No intervention |
| Ince 2017 | MM | Single session | 1 | Researcher / study team | Classical / instrumental music | Free-field playback | Clinical skill practice | Standard training (no music) |
| Indira 2018 | MM | 10 min | 60 | Researcher / study team | Indian classical instrumental music | Individual listening via headphones/earphones | NR | No music intervention |
| Kucukkaya 2024 | MM | 2 menstrual cycles | 8 | Researcher / study team | Music-based intervention | Individual listening via headphones/earphones | Menstrual period | No intervention / routine care |
| Lai 2008 | MM | 40 min | 2 | Researcher / study team | Classical / instrumental music | Free-field playback | Examination / OSCE | Silence (regular exam, no music) |
| Oner 2026 | MM | 20 min | 1 | Research team | MusiCure with nature sounds | Room playback; volume adjusted for comfort | Before ventrogluteal injection practice | Aromatherapy alone (incremental added-music contrast); no-treatment third arm not used for this contrast |
| Pellico 2012 | Music auditory training / active educational intervention | 2 hours | 1 | Research and clinical faculty with music expertise | Purpose-built pitch, rhythm, timbre, frequency and masking exercises | Group training using music, visual notation, synthesized and recorded body sounds | Physical-assessment and auscultation education, week 13 | Traditional assessment teaching only |
| Selcuk 2022 | Music medicine / passive listening | 29 min 32 s per day for 4 days | 4 | Research team; remote reminders | Occasio Adolore, purpose-composed relaxing music | Individual headphone listening via online audio link | Primary dysmenorrhea; 3 days before and first day of menstruation | No intervention; separate dark-chocolate third arm |
| Son 2019 | MM | 20 min | 1 | Researcher / study team | Classical / instrumental music | Free-field playback | NR | Aromatherapy alone (incremental added-music contrast); music-only and no-treatment arms not used for this contrast |
| Su 2026 | Music medicine / passive listening | 15 min before practice plus low-volume music throughout a 4-hour laboratory session | 1 | Research team | Acemashiran Turkish music | Computer loudspeaker in the skills laboratory | Peripheral intravenous catheter placement skills laboratory | Standard skill training without music |
| Summers 1990 | Music medicine / passive listening | Throughout a 2-hour course examination | 1 | Research team | Mind Body Tempo | Classroom playback at approximately 48-52 dB | Course examination | Comparable course examination without music |

## Table S4. RoB 2 judgments for randomized studies

| **Study** | **Variant** | **D1** | **Design-specific domain** | **D2** | **D3** | **D4** | **D5** | **Overall** | **Rationale** |
| --- | --- | --- | --- | --- | --- | --- | --- | --- | --- |
| Aksoy 2024 | Parallel | Some concerns | Not applicable | Some concerns | Some concerns | High | Some concerns | High | Sequence generation and concealment were insufficiently detailed and 60 were randomized but only 50 analyzed; state anxiety was unblinded self-report. |
| Borzoo 2025 | Parallel | Low | Not applicable | Low | Low | Low | Some concerns | Some concerns | Independent computerized randomization and opaque numbered cards were reported; objective vital signs were complete, but no prespecified public analysis plan was identified. |
| Cetin 2024 | Parallel | Low | Not applicable | Some concerns | High | High | Low | High | Thirty-nine of 168 randomized participants were excluded after randomization for potentially outcome-related reasons; pain and comfort were unblinded participant-reported outcomes. |
| Chen 2015 | Parallel | Low | Not applicable | Some concerns | Some concerns | High | Some concerns | High | Random assignment was reported, but deviations and attrition were incompletely documented; the principal mood outcome was unblinded self-report and no public analysis plan was found. |
| Contreras 2025 | Cluster | Some concerns | Some concerns | Some concerns | Some concerns | High | Some concerns | High | Recording groups were cluster-randomized, but sequence and cluster handling were incompletely described; seven of 96 randomized students declined, age differed between groups, clustering was not incorporated, and STAI was unblinded self-report. |
| Ergin 2025 | Parallel | Some concerns | Not applicable | Some concerns | Some concerns | High | Some concerns | High | Sequence/concealment and intervention deviations were not fully documented; the principal anxiety/comfort outcomes were unblinded self-report. |
| Erzincanli 2025 | Cluster | Some concerns | High | High | Low | High | Some concerns | High | Only two pre-existing classes were randomized, recruitment followed known class allocation, and clustering was ignored; state anxiety was unblinded and reporting emphasized within-group changes. |
| Eyuboglu 2021 | Parallel | Some concerns | Not applicable | Some concerns | Some concerns | High | Some concerns | High | Allocation concealment and deviation handling were unclear; the principal state-anxiety outcome was unblinded self-report and no prespecified analysis plan was available. |
| Gebhart 2020 | Parallel | Some concerns | Not applicable | Some concerns | High | High | Some concerns | High | Allocation concealment was unclear, 15 of 72 randomized participants lacked complete data, and state anxiety/stress were unblinded participant-reported outcomes. |
| Gosselin 2024 | Parallel | Some concerns | Not applicable | Some concerns | Low | High | Some concerns | High | Concealment and adherence details were limited; anxiety and self-efficacy were unblinded self-report, and performance was available only for a selected subset. |
| Inangil 2020a | Parallel | Low | Not applicable | Some concerns | Low | High | Some concerns | High | A computer-generated random order was used and outcome data were complete, but participants and providers were unblinded and the principal anxiety outcome was self-reported. |
| Inangil 2020b | Parallel | Low | Not applicable | Some concerns | Some concerns | High | Some concerns | High | Randomization was reported, but adherence and missing-data handling were incompletely described; anxiety was unblinded self-report with no public analysis plan. |
| Indira 2018 | Parallel | Some concerns | Not applicable | Some concerns | Low | High | Some concerns | High | Lottery allocation supported randomization but concealment and adherence were unclear; baseline study-habit imbalances and an unblinded self-rated performance scale increased bias concerns. |
| Kucukkaya 2024 | Parallel | Low | Not applicable | Some concerns | Low | High | Low | High | Randomization and completeness were acceptable, but pain and perceived stress were unblinded participant-reported outcomes. |
| Lai 2008 | Crossover | Some concerns | Some concerns | Low | Low | High | Some concerns | High | Sequence balance, concealment and period/carryover modeling were incompletely reported; anxiety was unblinded self-report and paired variance was unavailable. |
| Oner 2026 | Parallel | Low | Not applicable | Some concerns | Low | High | Low | High | Randomization and completeness were acceptable, but the principal anxiety/self-esteem outcomes were unblinded self-report. |
| Pellico 2012 | Parallel | Some concerns | Not applicable | Some concerns | Some concerns | Low | Some concerns | Some concerns | Sequence generation was adequate but concealment was not described; nine of 78 lacked posttest data and LOCF was used. The auditory test was objective. |
| Selcuk 2022 | Parallel | Some concerns | Not applicable | Some concerns | Some concerns | High | Some concerns | High | Lottery-based assignment was described but concealment and three-arm sequence details were incomplete; six participants discontinued and the ITT imputation method was unclear; pain and anxiety were unblinded self-report. |
| Son 2019 | Parallel | Low | Not applicable | Low | Some concerns | High | Some concerns | High | Independent randomization with opaque envelopes was reported; nine of 107 randomized participants were excluded, and unblinded participant-reported anxiety creates high measurement risk. |
| Su 2026 | Parallel | High | Not applicable | Some concerns | Low | High | Some concerns | High | The report states simple randomization but does not describe sequence generation or concealment and presents an unexplained 144-to-60 flow; anxiety was unblinded self-report and registration appears later than study conduct. |

## Table S5. ROBINS-I judgments for nonrandomized studies

| **Study** | **D1** | **D2** | **D3** | **D4** | **D5** | **D6** | **D7** | **Overall** | **Rationale** |
| --- | --- | --- | --- | --- | --- | --- | --- | --- | --- |
| Bittman 2004 | Serious | Low | Low | Low | Moderate | Serious | Moderate | Serious | The crossover CCT did not adequately model period, historical or carryover effects. Completion was incompletely reported, and MBI/POMS were unblinded self-report without a public analysis plan. |
| Evangelista 2017 | Serious | Moderate | Low | Low | Low | Serious | Moderate | Serious | Pre-existing course sections and class context were confounded with exposure and not adjusted. DASS outcomes were unblinded self-report; no protocol or analysis plan was available. |
| Ince 2017 | Serious | Moderate | Low | Low | Low | Serious | Moderate | Serious | Selected study groups determined exposure without adequate adjustment for class, teacher, sex imbalance or baseline anxiety. State anxiety and skill assessment were susceptible to knowledge of intervention. |
| Summers 1990 | Serious | Moderate | Low | Low | Low | Serious | Moderate | Serious | Two intact course cohorts determined treatment, leaving course, instructor and examination confounding. Anxiety was unblinded self-report and pulse was self-measured; no prespecified protocol was identified. |

## Table S6. Principal postintervention anxiety dataset

| **Study** | **Context** | **n music** | **n control** | **Hedges g** | **95% CI** | **REML weight** | **Basis** | **Source** |
| --- | --- | --- | --- | --- | --- | --- | --- | --- |
| Aksoy 2024 | Examination / OSCE | 18 | 16 | -0.800 | -1.501 to -0.099 | 8.0% | Unadjusted postintervention arm means | Primary report Table 2 |
| Ergin 2025 | Simulation | 40 | 37 | 0.220 | -0.229 to 0.668 | 11.8% | Unadjusted postintervention arm means | Primary report Table 2 |
| Eyuboglu 2021 | Examination / OSCE | 61 | 64 | 0.052 | -0.299 to 0.403 | 13.5% | Unadjusted postintervention arm means | Primary report Table 1 |
| Gosselin 2024 | Simulation | 51 | 51 | -0.412 | -0.804 to -0.020 | 12.8% | Unadjusted postintervention arm means | Primary report Results |
| Inangil 2020b | Before first clinical practice | 30 | 28 | -0.564 | -1.090 to -0.039 | 10.5% | Unadjusted postintervention arm means | Primary report Table 2 |
| Oner 2026 | Before ventrogluteal injection practice | 34 | 34 | 0.245 | -0.233 to 0.722 | 11.3% | Unadjusted postintervention arm means | Primary report Table 3 |
| Selcuk 2022 | Primary dysmenorrhea; 3 days before and first day of menstruation | 30 | 30 | -0.390 | -0.901 to 0.121 | 10.7% | Unadjusted postintervention arm means | Primary report Table 4 |
| Son 2019 | NR | 34 | 32 | -0.575 | -1.068 to -0.082 | 11.0% | Unadjusted postintervention arm means | Primary report Table 3 |
| Su 2026 | Peripheral intravenous catheter placement skills laboratory | 30 | 30 | -0.845 | -1.374 to -0.316 | 10.4% | Unadjusted postintervention arm means | Primary report Table 3 |

## Table S7. Full-text exclusions among 39 complete documents

| **Record** | **Title** | **Primary reason** |
| --- | --- | --- |
| S0035 | Effectiveness of music therapy and progressive muscle relaxation in reducing stress before exams and improving academic performance in Nursing students: A randomized trial. | Music intervention ineligible/not isolatable |
| S0040 | Effects of a Cognitive-Behavioral-Integrated Musical Rhythms Intervention on Anger: A Randomized, Single-Blind Factorial Trial. | No eligible outcome |
| S0065 | Impact of a Media-Based Music Therapy Intervention on Anxiety, Burnout, and Clinical Performance in Nursing Students. | Music intervention ineligible/not isolatable |
| S0069 | Romantic Song Lyrics Used to Facilitate Nursing Students' Comprehension of Qualitative Data Analysis. | Music intervention ineligible/not isolatable |
| S0079 | Looking Is Not Seeing and Listening Is Not Hearing: A Replication Study With Accelerated BSN Students. | No eligible comparator |
| S0097 | Harnessing the Power of Song to Teach Qualitative Thematic Analysis. | Music intervention ineligible/not isolatable |
| S0122 | Music for Anxiety Reduction and Performance Enhancement in Nursing Simulation. | Music intervention ineligible/not isolatable |
| S0143 | Stress Management Intervention for Nursing Students Working Night Shifts. | Music intervention ineligible/not isolatable |
| S0185 | Lyrics for Learning: Using Song to Improve Knowledge Retention. | Music intervention ineligible/not isolatable |
| S0187 | Artwork and music: Innovative approaches to physical assessment. | No eligible comparator |
| S0211 | Music Lyrics: A Student-Centered Approach to Engaging Online Learning. | Music intervention ineligible/not isolatable |
| S0378 | Effect of Music Therapy on Sleep Quality | Music intervention ineligible/not isolatable |
| S0392 | ARTS IN HEALTH: USING THE ARTS IN UNDERGRADUATE NURSING EDUCATION TO FOSTER CRITICAL THINKING SKILLS | Music intervention ineligible/not isolatable |

## Table S8. Principal and sensitivity analyses

| **Analysis** | **k** | **Estimate (95% CI)** | **I²** | **τ²** | **HK 95% CI** | **95% prediction interval** |
| --- | --- | --- | --- | --- | --- | --- |
| Principal postintervention model | 9 | -0.309 (-0.577 to -0.040) | 64.3% | 0.107 | -0.625 to 0.008 | -1.147 to 0.530 |
| Passive listening; active music therapy excluded | 8 | -0.365 (-0.654 to -0.076) | 62.4% | 0.107 | -0.712 to -0.017 | -1.244 to 0.514 |
| Add-on contrasts excluded | 7 | -0.350 (-0.649 to -0.051) | 63.5% | 0.101 | -0.721 to 0.021 | -1.256 to 0.557 |
| Standalone passive listening | 6 | -0.433 (-0.748 to -0.117) | 57.2% | 0.088 | -0.840 to -0.025 | -1.369 to 0.504 |
| Trial at high D1 risk excluded | 8 | -0.242 (-0.505 to 0.022) | 59.3% | 0.084 | -0.564 to 0.081 | -1.026 to 0.542 |
| Change scores, r=0.25 | 8 | -0.377 (-0.575 to -0.179) | 12.4% | 0.020 | -0.588 to -0.166 | -0.804 to 0.050 |
| Change scores, r=0.50 | 8 | -0.431 (-0.641 to -0.221) | 24.3% | 0.029 | -0.656 to -0.207 | -0.924 to 0.061 |
| Change scores, r=0.75 | 8 | -0.554 (-0.799 to -0.309) | 49.9% | 0.060 | -0.825 to -0.282 | -1.226 to 0.119 |

## Table S9. Leave-one-out sensitivity analysis

| **Omitted study** | **k** | **Wald estimate (95% CI)** | **HK 95% CI** | **I²** |
| --- | --- | --- | --- | --- |
| Aksoy 2024 | 8 | -0.265 (-0.542 to 0.011) | -0.601 to 0.070 | 64.9% |
| Ergin 2025 | 8 | -0.375 (-0.643 to -0.107) | -0.696 to -0.054 | 59.8% |
| Eyuboglu 2021 | 8 | -0.365 (-0.654 to -0.076) | -0.712 to -0.017 | 62.4% |
| Gosselin 2024 | 8 | -0.298 (-0.605 to 0.009) | -0.668 to 0.072 | 67.7% |
| Inangil 2020b | 8 | -0.281 (-0.574 to 0.013) | -0.639 to 0.078 | 66.6% |
| Oner 2026 | 8 | -0.374 (-0.642 to -0.107) | -0.692 to -0.057 | 60.2% |
| Selcuk 2022 | 8 | -0.303 (-0.605 to 0.000) | -0.670 to 0.065 | 68.3% |
| Son 2019 | 8 | -0.277 (-0.571 to 0.016) | -0.635 to 0.080 | 66.1% |
| Su 2026 | 8 | -0.242 (-0.505 to 0.022) | -0.564 to 0.081 | 59.3% |

## Table S10. Directly verified study-specific secondary effects

| **Study** | **Outcome** | **Context** | **Measure** | **Effect (95% CI)** | **Unit** | **Direct source** | **Basis / caution** |
| --- | --- | --- | --- | --- | --- | --- | --- |
| Gosselin 2024 | self efficacy | Simulation | SMD_Hedges_g | 0.73 (0.33 to 1.13) | SD units | Calculated from reported postintervention means and SDs | Unadjusted postintervention score; Clinical and Simulation General Self-Efficacy Scale. |
| Gosselin 2024 | simulation performance | Simulation | SMD_Hedges_g | 1.71 (0.67 to 2.75) | SD units | Calculated from reported postintervention means and SDs | Unadjusted postintervention score in selected subset; Instructor rating 0-100; selected subset of 20 participants, so selection bias is possible. |
| Kucukkaya 2024 | pain | Dysmenorrhea | MD | -5.43 (-6.30 to -4.56) | scale points | Figure 4C reconstructed | Change score; r=0.50 for change SD; Figure values rounded to two decimals |
| Kucukkaya 2024 | stress | Dysmenorrhea | SMD_Hedges_g | -6.94 (-8.26 to -5.62) | SD units | Figure 4B reconstructed | Change score; r=0.50 for change SD; Figure values rounded to two decimals |
| Oner 2026 | diastolic blood pressure | Clinical skills | MD | 0.54 (-4.29 to 5.37) | mmHg | Corrected from primary report | Change score; r=0.50 for change SD; AM versus aromatherapy alone; change SD calculated with pre-post r=0.50 |
| Oner 2026 | heart rate | Clinical skills | MD | -2.32 (-9.65 to 5.01) | beats/min | Corrected from primary report | Change score; r=0.50 for change SD; AM versus aromatherapy alone; change SD calculated with pre-post r=0.50 |
| Oner 2026 | systolic blood pressure | Clinical skills | MD | -2.50 (-8.19 to 3.19) | mmHg | Corrected from primary report | Change score; r=0.50 for change SD; AM versus aromatherapy alone; change SD calculated with pre-post r=0.50 |
| Selcuk 2022 | pain | Primary dysmenorrhea | MD | -0.30 (-1.38 to 0.78) | VAS points | Primary report Table 3 | Unadjusted postintervention arm means; Reported ITT table used. Change-score SD was unavailable. |
| Su 2026 | diastolic blood pressure | PIVC skills laboratory | MD | 5.66 (1.72 to 9.60) | mmHg | Primary report Table 3 | Unadjusted postintervention arm means |
| Su 2026 | heart rate | PIVC skills laboratory | MD | 5.24 (-0.93 to 11.41) | beats/min | Primary report Table 3 | Unadjusted postintervention arm means |
| Su 2026 | oxygen saturation | PIVC skills laboratory | MD | -0.83 (-1.63 to -0.03) | percentage points | Primary report Table 3 | Unadjusted postintervention arm means |
| Su 2026 | respiratory rate | PIVC skills laboratory | MD | 0.04 (-1.04 to 1.12) | breaths/min | Primary report Table 3 | Unadjusted postintervention arm means |
| Su 2026 | self confidence | PIVC skills laboratory | MD | 0.23 (-0.65 to 1.11) | 0-10 points | Primary report Table 3 | Unadjusted postintervention arm means |
| Su 2026 | simulation performance | PIVC skills laboratory | MD | 0.46 (-2.35 to 3.27) | PIVC checklist points | Primary report Table 2 | Unadjusted postintervention arm means |
| Su 2026 | systolic blood pressure | PIVC skills laboratory | MD | 8.00 (2.68 to 13.32) | mmHg | Primary report Table 3 | Unadjusted postintervention arm means |
| Summers 1990 | heart rate | Course examination | MD | -7.33 (-16.75 to 2.09) | beats/min | Primary report Table 5 | Unadjusted postintervention arm means; Pulse was self-measured; nonrandom intact course cohorts. |
| Summers 1990 | test anxiety | Course examination | SMD_Hedges_g | 0.03 (-0.56 to 0.61) | SD units | Primary report Table 4 | Unadjusted postintervention arm means; Nonrandom intact course cohorts; unadjusted post-test contrast. |
| Contreras 2025 | academic performance pass | First-semester skills competency | Risk ratio | 1.13 (0.85 to 1.50) | Pass | Primary dissertation Table 5.4 | Unadjusted pass proportion; clustering not incorporated; Music 36/49 passed; control 26/40 passed. The report gave chi-square p=0.387. The risk-ratio CI is descriptive because clustering was not incorporated. |
| Aksoy 2024 | intramuscular injection skill | Immediate intramuscular-injection skill examination | SMD_Hedges_g | 0.26 (-0.41 to 0.94) | SD units | Primary report Table 4 | Unadjusted postintervention arm means; 29-step checklist converted to a 100-point score. |
| Evangelista 2017 | knowledge change | After a three-hour lecture | SMD_Hedges_g | -0.08 (-0.68 to 0.53) | SD units | Primary report Results and Table 2 | Reported pre-to-post change means and SDs; Researcher-developed 15-item factual-recall quiz; content reviewed by two faculty members. |
| Eyuboglu 2021 | osce average score | Immediately after the first OSCE | SMD_Hedges_g | -0.05 (-0.40 to 0.31) | SD units | Primary report Table 3 | Unadjusted postintervention arm means; Average of intramuscular injection, IV fluid therapy, and tracheostomy aspiration scores. |
| Son 2019 | fundamental nursing skill performance | Immediate Foley-catheterization performance test | SMD_Hedges_g | 0.82 (0.32 to 1.33) | SD units | Primary report Table 3 | Aromatherapy plus music versus aromatherapy alone; blinded assessor; 100-point structured scorecard. The contrast estimates the incremental contribution of music when both groups received aromatherapy. |

## Table S11. Structured educational-outcome evidence

| **Study** | **Sample** | **Construct / measure** | **Timing** | **Between-group result** | **Validity / interpretation** | **Risk of bias** |
| --- | --- | --- | --- | --- | --- | --- |
| Aksoy 2024 | Music 18; control 16 | IM-injection skill; 29-step checklist, 0–100 | Immediate skill exam | g 0.26 (95% CI −0.41 to 0.94) | No clear between-group difference; checklist derived from literature | High |
| Contreras 2025 | Music 49; control 40 | Semester skills competency pass/fail | End of semester | RR 1.13 (95% CI 0.85 to 1.50); report p=0.387 | Recording groups randomized; CI is descriptive because clustering was not incorporated | High |
| Evangelista 2017 | Music 21; control 21 | Knowledge; researcher-developed 15-item quiz | After a three-hour lecture | Change-score g −0.08 (95% CI −0.68 to 0.53) | Content reviewed by two faculty members; no clear effect | Serious (ROBINS-I) |
| Eyüboğlu 2021 | Music therapy 61; control 64 | Mean of three OSCE skills | Immediately after first OSCE | g −0.05 (95% CI −0.40 to 0.31) | No clear difference; three component scores also nonsignificant | High |
| Gosselin 2024 | Music 51; control 51; performance subset 10/10 | Clinical and Simulation General Self-Efficacy; instructor performance rating | Immediate simulation test | Self-efficacy g 0.73 (0.33 to 1.13); performance g 1.71 (0.67 to 2.75) | Performance estimate came from a selected 20-participant subset | High |
| İnce 2017 | Music 34; control 39 | Blood-draw procedural steps | Immediate laboratory practice | No single composite effect reported | Step-level correct/wrong/forgotten frequencies only | Serious (ROBINS-I) |
| Indira 2018 | Music 91; control 100 | 19-item Academic Performance Rating Scale | 30 days | Nine of 19 item comparisons p<0.05; composite arm means/SDs unavailable | Researcher-developed measure; item-level multiplicity; no analysis-ready composite | High |
| Pellico 2012 | Randomized 78; posttest n=69 | Organ identification and diagnosis of 25 body sounds | Ten days after training; week 14 | Diagnosis change +9.5 vs +3.5, interaction p=0.001; adjusted organ-identification p=0.005 | Baseline imbalance for organ identification; Poisson adjustment reported | Some concerns |
| Son 2019 | Music plus aromatherapy 34; aromatherapy 32 | Foley-catheterization score, 0–100 | Immediate blinded performance test | g 0.82 (95% CI 0.32 to 1.33) | Incremental music contrast; both groups received aromatherapy | High |
| Su 2026 | Music 30; control 30 | PIVC checklist; 0–10 self-confidence | Immediate skills laboratory | Skill MD 0.46 (−2.35 to 3.27); self-confidence MD 0.23 (−0.65 to 1.11) | No clear between-group effect | High |

## Table S12. Study-level overlap with the 2026 health-sciences meta-analysis

| **Report / study** | **Current review** | **2026 review** | **Status** |
| --- | --- | --- | --- |
| Aksoy 2024 | Yes | No | Current review only |
| Bittman 2004 | Yes | No | Current review only |
| Borzoo 2025 | Yes | Yes | Shared |
| Cetin 2024 | Yes | Yes | Shared |
| Chen 2015 | Yes | Yes | Shared |
| Contreras 2025 | Yes | Yes | Shared |
| Dag 2025 | No | Yes | 2026 meta-analysis only |
| Dogan thesis 2025 | No | Yes | 2026 meta-analysis only |
| Ekitli 2022 | No | Yes | 2026 meta-analysis only |
| Ergin 2025 | Yes | No | Current review only |
| Erzincanli 2025 | Yes | Yes | Shared |
| Evangelista 2017 | Yes | Yes | Shared |
| Eyuboglu 2021 | Yes | Yes | Shared |
| Fuson 2012 | No | Yes | 2026 meta-analysis only |
| Gebhart 2020 | Yes | Yes | Shared |
| Gosselin 2016 | No | Yes | 2026 meta-analysis only |
| Gosselin 2024 | Yes | Yes | Shared |
| Inangil 2020a | Yes | Yes | Shared |
| Inangil 2020b | Yes | Yes | Shared |
| Ince 2017 | Yes | Yes | Shared |
| Indira 2018 | Yes | Yes | Shared |
| Kavurmaci 2020 | No | Yes | 2026 meta-analysis only |
| Kucukkaya 2024 | Yes | Yes | Shared |
| Lai 2008 | Yes | Yes | Shared |
| Mojarrab 2020 | No | Yes | 2026 meta-analysis only |
| Oner 2026 | Yes | No | Current review only |
| Pellico 2012 | Yes | No | Current review only |
| Selcuk 2022 | Yes | No | Current review only |
| Son 2019 | Yes | No | Current review only |
| Su 2026 | Yes | No | Current review only |
| Summers 1990 | Yes | No | Current review only |
| Tastan 2017 | No | Yes | 2026 meta-analysis only |
| Yoo 2013 | No | Yes | 2026 meta-analysis only |

Overlap summary: 15 of 24 reports were shared; nine were unique to each review. Overlap within each review was 62.5%; Jaccard overlap was 15/33 = 45.5%.

## Table S13. Reports sought but not retrieved as complete documents

| **Record** | **Title** | **DOI** | **Record type** |
| --- | --- | --- | --- |
| S0101 | [A teaching technique for nursing students to learn drugs by singing songs with drug rhyames]. | No DOI identified | bibliographic |
| S0128 | Building collaborative practice through interprofessional education: music therapy and nursing students engaged in collaborative research. | 10.1080/08098131.2016.1179981 | bibliographic |
| S0148 | 2008 SNRS abstracts -- W - Z. | No DOI identified | bibliographic |
| S0151 | Song as a mnemonic teaching strategy to enhance memory and higher-order cognition in an undergraduate nursing pharmacology course. | No DOI identified | bibliographic |
| S0179 | The power of music. | No DOI identified | bibliographic |
| S0208 | A preliminary study of music effects on nursing students' test anxiety. | 10.20467/1091-5710.10.2.85 | bibliographic |
| S0238 | The power of music. | 10.1891/1078-4535.16.2.84 | bibliographic |
| S0244 | Music That Moves You. | No DOI identified | bibliographic |
| S0245 | The Effect of Music Therapy and the 4-7-8 Breathing Exercise Technique on Anxiety Management Among Nursing Students During IV Drug Administration Education:Randomized Controled Trial | No DOI identified | bibliographic |
| S0250 | The Effects of Music Therapy and Aromatherapy Interventions on Pulse Rate, Blood Pressure, Anxiety, and Self-Esteem | No DOI identified | bibliographic |
| S0254 | Effects of Mandala Coloring and Music Activity on Future Anxiety and Subjective Well-Being in Nursing Students: A Randomized Controlled Trial | No DOI identified | bibliographic |
| S0263 | The Effect of Listening to Music and Drawing on the Coping With Dysmenorrhea of Nursing Students Experiencing Dysmenorrhea | No DOI identified | bibliographic |
| S0264 | The Effect of Progressive Muscle Relaxation and Deep Breathing Exercises Accompanied by Music on the Stress Levels of Nursing Students and Their Bio-psycho-social Responses to Stress | No DOI identified | bibliographic |
| S0267 | The Effect of Music on Nursing Students' Skills, Anxiety, Self-Confidence Perception, Vital Sings | No DOI identified | bibliographic |
| S0268 | Comparison of the Effect of Hegu Point Ice Massage and Music on Pain and Comfort Levels in Dysmenorrhea | No DOI identified | registry |
| S0271 | The Effect of Dark Chocolate and Music on Primary Dysmenorrhea | No DOI identified | registry |
| S0275 | The Effect of Gross' Process Model of Emotion Regulation Integrated in Musical Rhythm Interventions on Student Nurses' Anger Expressions and States | No DOI identified | bibliographic |
| S0279 | The Effects of Combined Music Care With Sleep Management Network on Sleep Quality, Sleep Knowledge, and Sleep Hygiene in Nursing Students | No DOI identified | bibliographic |
| S0301 | Survey of holy quran voice influence on nursing school students after Bam earth quake | 10.1016/s1876-2018(11)60170-6 | bibliographic |
| S0308 | Effects of Aromatherapy Combined with Music Therapy on Test Anxiety, State Anxiety, Stress, and Fundamental Nursing Skills Performance in Nursing Students | No DOI identified | bibliographic |
| S0327 | An Empirical Study on the Intervention with Traditional Chinese Medicine Five-Element Music Therapy on Depressive Symptoms in College Students Majoring in Nursing: a Comparison of the Effects of Its Three Tempos | No DOI identified | bibliographic |
| S0329 | Effects of self-selected music listening and mechanical chair massage on test anxiety in undergraduate pre-licensure associate of applied science nursing students. | No DOI identified | bibliographic |
| S0368 | Exploring Mental Health Issues With Psychiatric Mental Health (PMH) Nursing Students Through the Use of Contemporary Music | 10.1177/10783903241308917 | bibliographic |

# Supplementary figures

Figure S1. ROBINS-I traffic-light plot for four nonrandomized controlled studies.

Figure S2. Principal and sensitivity estimates for acute or short-term anxiety. Negative values favor music-based intervention.

Figure S3. Leave-one-out analysis of the principal postintervention anxiety model.
